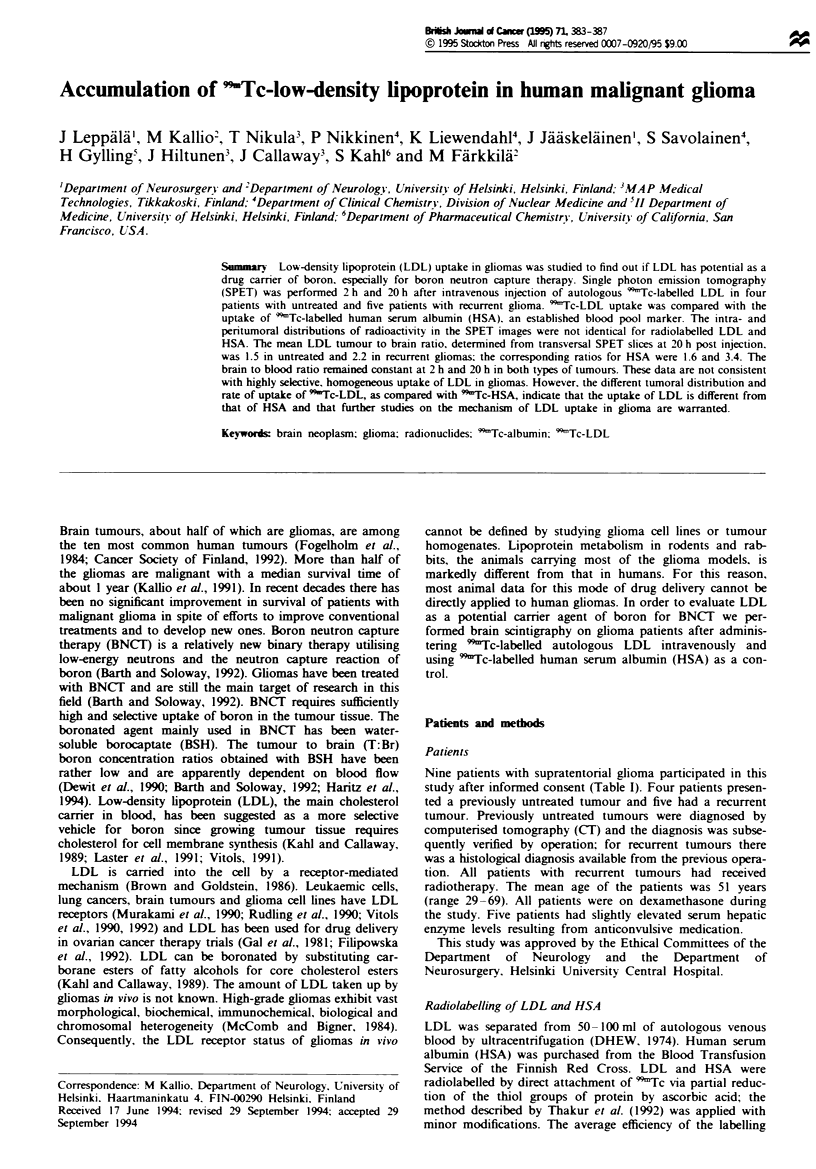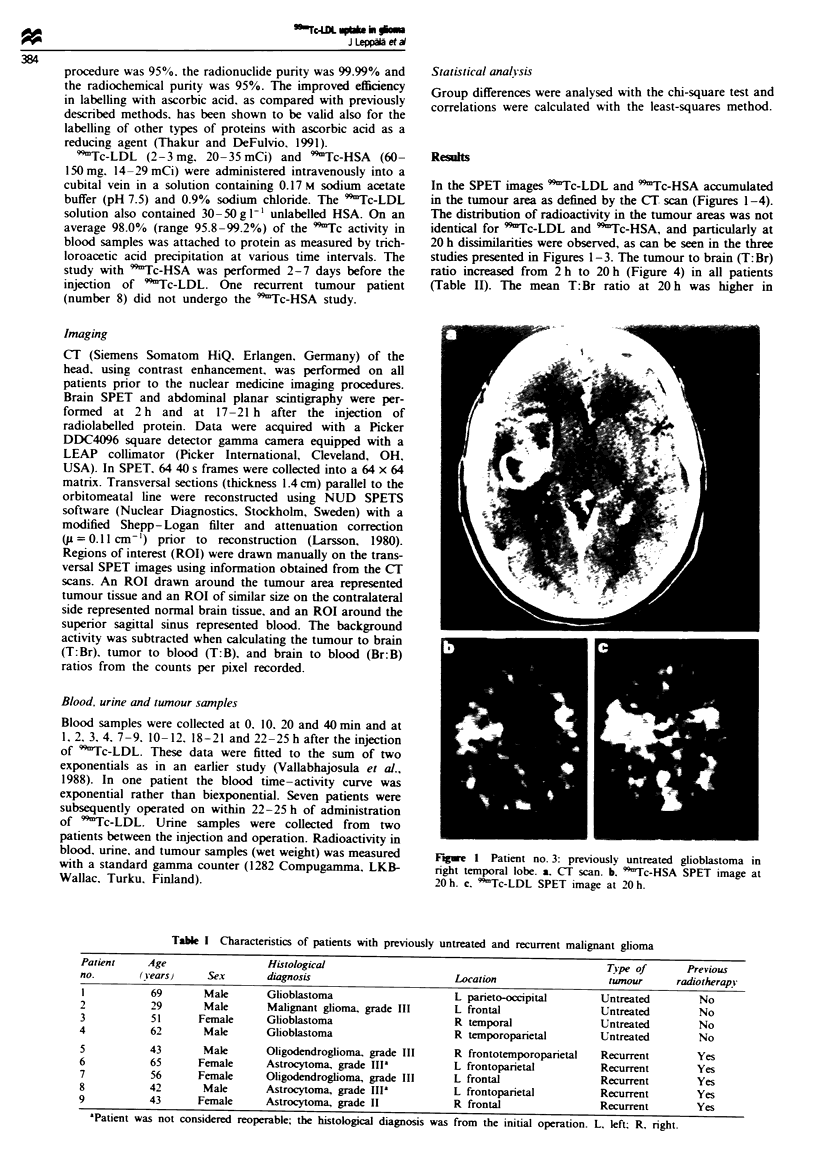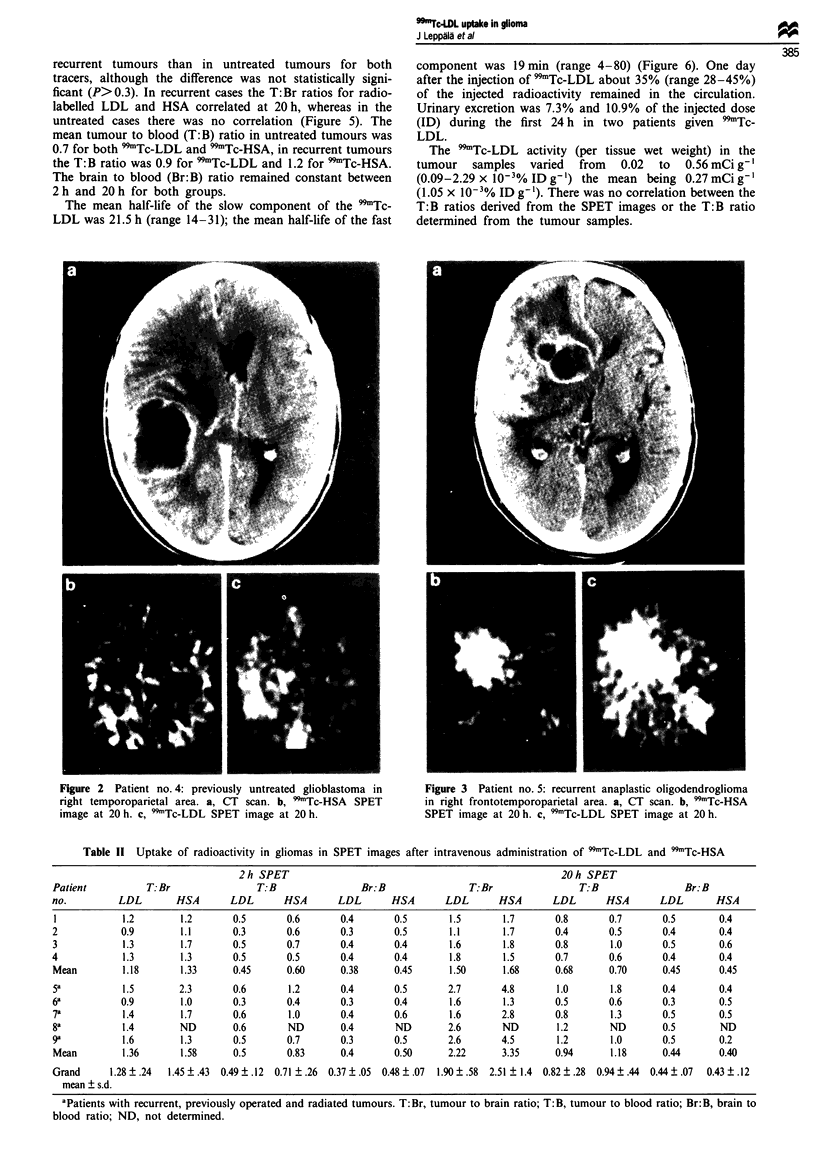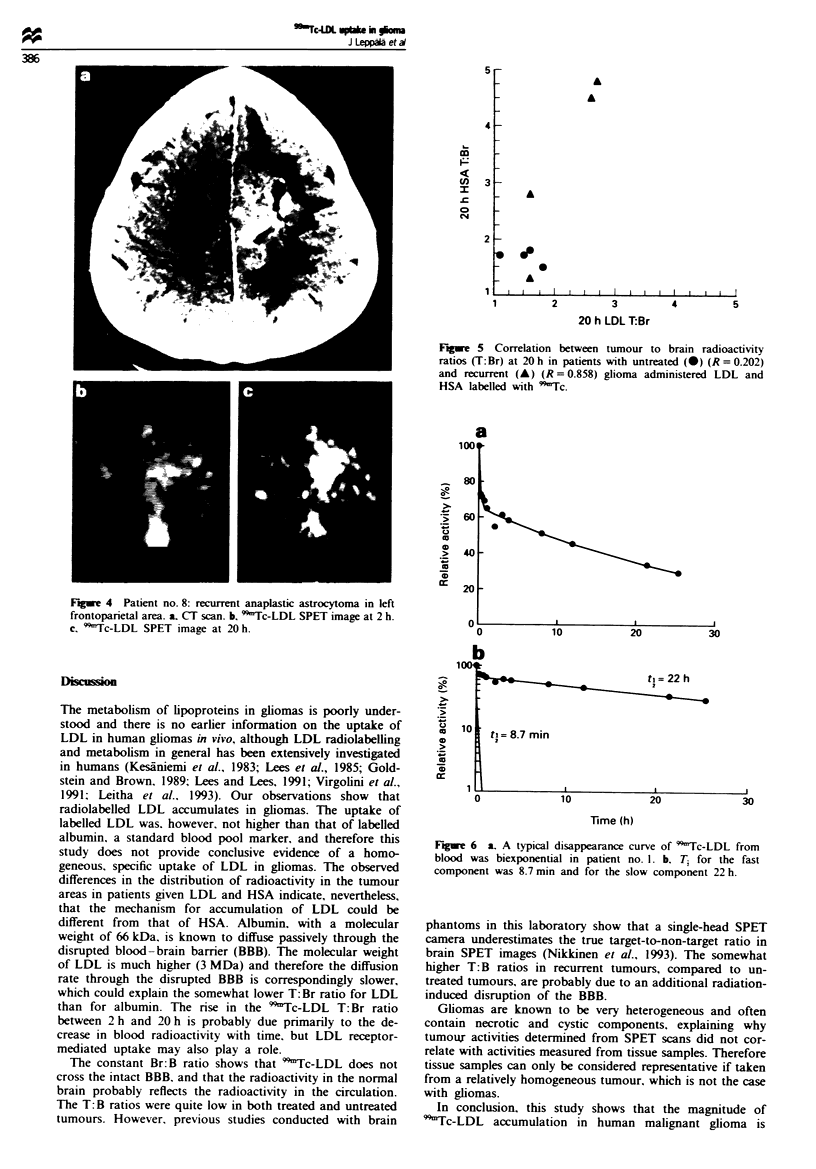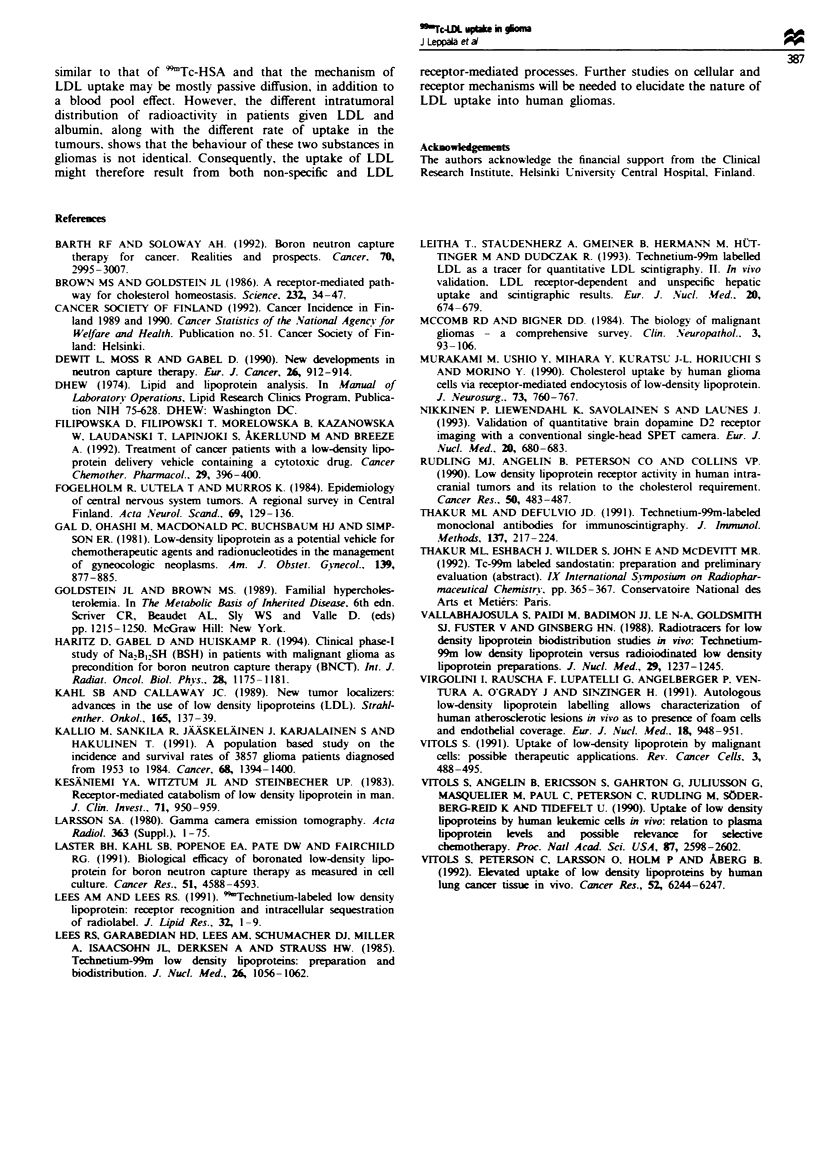# Accumulation of 99mTc-low-density lipoprotein in human malignant glioma.

**DOI:** 10.1038/bjc.1995.78

**Published:** 1995-02

**Authors:** J. Leppälä, M. Kallio, T. Nikula, P. Nikkinen, K. Liewendahl, J. Jääskeläinen, S. Savolainen, H. Gylling, J. Hiltunen, J. Callaway

**Affiliations:** Department of Neurosurgery, University of Helsinki, Finland.

## Abstract

**Images:**


British Journal of Cancer (1995) 71, 383-387

© 1995 Stockton Press All rights reserved 0007-0920/95 $9.00

### Accumulation of $^{99m}$Tc-low-density lipoprotein in human malignant glioma

J Leppälä[1], M Kallio[2], T Nikula[3], P Nikkinen[4], K Liewendahl[4], J Jääskeläinen[1], S Savolainen[4], H Gylling[5], J Hiltunen[3], J Callaway[3], S Kahl[6] and M Färkkilä[2]

*[1]Department of Neurosurgery and [2]Department of Neurology, University of Helsinki, Helsinki, Finland; [3]MAP Medical Technologies, Tikkakoski, Finland; [4]Department of Clinical Chemistry, Division of Nuclear Medicine and [5]II Department of Medicine, University of Helsinki, Helsinki, Finland; [6]Department of Pharmaceutical Chemistry, University of California, San Francisco, USA.*

**Summary** Low-density lipoprotein (LDL) uptake in gliomas was studied to find out if LDL has potential as a drug carrier of boron, especially for boron neutron capture therapy. Single photon emission tomography (SPET) was performed 2 h and 20 h after intravenous injection of autologous $^{99m}$Tc-labelled LDL in four patients with untreated and five patients with recurrent glioma. $^{99m}$Tc-LDL uptake was compared with the uptake of $^{99m}$Tc-labelled human serum albumin (HSA), an established blood pool marker. The intra- and peritumoral distributions of radioactivity in the SPET images were not identical for radiolabelled LDL and HSA. The mean LDL tumour to brain ratio, determined from transversal SPET slices at 20 h post injection, was 1.5 in untreated and 2.2 in recurrent gliomas; the corresponding ratios for HSA were 1.6 and 3.4. The brain to blood ratio remained constant at 2 h and 20 h in both types of tumours. These data are not consistent with highly selective, homogeneous uptake of LDL in gliomas. However, the different tumoral distribution and rate of uptake of $^{99m}$Tc-LDL, as compared with $^{99m}$Tc-HSA, indicate that the uptake of LDL is different from that of HSA and that further studies on the mechanism of LDL uptake in glioma are warranted.

**Keywords:** brain neoplasm; glioma; radionuclides; $^{99m}$Tc-albumin; $^{99m}$Tc-LDL

Brain tumours, about half of which are gliomas, are among the ten most common human tumours (Fogelholm *et al.*, 1984; Cancer Society of Finland, 1992). More than half of the gliomas are malignant with a median survival time of about 1 year (Kallio *et al.*, 1991). In recent decades there has been no significant improvement in survival of patients with malignant glioma in spite of efforts to improve conventional treatments and to develop new ones. Boron neutron capture therapy (BNCT) is a relatively new binary therapy utilising low-energy neutrons and the neutron capture reaction of boron (Barth and Soloway, 1992). Gliomas have been treated with BNCT and are still the main target of research in this field (Barth and Soloway, 1992). BNCT requires sufficiently high and selective uptake of boron in the tumour tissue. The boronated agent mainly used in BNCT has been water-soluble borocaptate (BSH). The tumour to brain (T:Br) boron concentration ratios obtained with BSH have been rather low and are apparently dependent on blood flow (Dewit *et al.*, 1990; Barth and Soloway, 1992; Haritz *et al.*, 1994). Low-density lipoprotein (LDL), the main cholesterol carrier in blood, has been suggested as a more selective vehicle for boron since growing tumour tissue requires cholesterol for cell membrane synthesis (Kahl and Callaway, 1989; Laster *et al.*, 1991; Vitols, 1991).

LDL is carried into the cell by a receptor-mediated mechanism (Brown and Goldstein, 1986). Leukaemic cells, lung cancers, brain tumours and glioma cell lines have LDL receptors (Murakami *et al.*, 1990; Rudling *et al.*, 1990; Vitols *et al.*, 1990, 1992) and LDL has been used for drug delivery in ovarian cancer therapy trials (Gal *et al.*, 1981; Filipowska *et al.*, 1992). LDL can be boronated by substituting carborane esters of fatty alcohols for core cholesterol esters (Kahl and Callaway, 1989). The amount of LDL taken up by gliomas *in vivo* is not known. High-grade gliomas exhibit vast morphological, biochemical, immunochemical, biological and chromosomal heterogeneity (McComb and Bigner, 1984). Consequently, the LDL receptor status of gliomas *in vivo* cannot be defined by studying glioma cell lines or tumour homogenates. Lipoprotein metabolism in rodents and rabbits, the animals carrying most of the glioma models, is markedly different from that in humans. For this reason, most animal data for this mode of drug delivery cannot be directly applied to human gliomas. In order to evaluate LDL as a potential carrier agent of boron for BNCT we performed brain scintigraphy on glioma patients after administering $^{99m}$Tc-labelled autologous LDL intravenously and using $^{99m}$Tc-labelled human serum albumin (HSA) as a control.

Correspondence: M Kallio, Department of Neurology, University of Helsinki, Haartmaninkatu 4, FIN-00290 Helsinki, Finland
Received 17 June 1994; revised 29 September 1994; accepted 29 September 1994

### Patients and methods

### *Patients*

Nine patients with supratentorial glioma participated in this study after informed consent (Table I). Four patients presented a previously untreated tumour and five had a recurrent tumour. Previously untreated tumours were diagnosed by computerised tomography (CT) and the diagnosis was subsequently verified by operation; for recurrent tumours there was a histological diagnosis available from the previous operation. All patients with recurrent tumours had received radiotherapy. The mean age of the patients was 51 years (range 29-69). All patients were on dexamethasone during the study. Five patients had slightly elevated serum hepatic enzyme levels resulting from anticonvulsive medication.

This study was approved by the Ethical Committees of the Department of Neurology and the Department of Neurosurgery, Helsinki University Central Hospital.

### *Radiolabelling of LDL and HSA*

LDL was separated from 50-100 ml of autologous venous blood by ultracentrifugation (DHEW, 1974). Human serum albumin (HSA) was purchased from the Blood Transfusion Service of the Finnish Red Cross. LDL and HSA were radiolabelled by direct attachment of $^{99m}$Tc via partial reduction of the thiol groups of protein by ascorbic acid; the method described by Thakur *et al.* (1992) was applied with minor modifications. The average efficiency of the labelling procedure was 95%, the radionuclide purity was 99.99% and the radiochemical purity was 95%. The improved efficiency in labelling with ascorbic acid, as compared with previously described methods, has been shown to be valid also for the labelling of other types of proteins with ascorbic acid as a reducing agent (Thakur and DeFulvio, 1991).

$^{99m}$Tc-LDL (2–3 mg, 20–35 mCi) and $^{99m}$Tc-HSA (60–150 mg, 14–29 mCi) were administered intravenously into a cubital vein in a solution containing 0.17 M sodium acetate buffer (pH 7.5) and 0.9% sodium chloride. The $^{99m}$Tc-LDL solution also contained 30–50 g $l^{-1}$ unlabelled HSA. On an average 98.0% (range 95.8–99.2%) of the $^{99m}$Tc activity in blood samples was attached to protein as measured by trichloroacetic acid precipitation at various time intervals. The study with $^{99m}$Tc-HSA was performed 2–7 days before the injection of $^{99m}$Tc-LDL. One recurrent tumour patient (number 8) did not undergo the $^{99m}$Tc-HSA study.

*Imaging*

CT (Siemens Somatom HiQ, Erlangen, Germany) of the head, using contrast enhancement, was performed on all patients prior to the nuclear medicine imaging procedures. Brain SPET and abdominal planar scintigraphy were performed at 2 h and at 17–21 h after the injection of radiolabelled protein. Data were acquired with a Picker DDC4096 square detector gamma camera equipped with a LEAP collimator (Picker International, Cleveland, OH, USA). In SPET, 64 40 s frames were collected into a 64 × 64 matrix. Transversal sections (thickness 1.4 cm) parallel to the orbitomeatal line were reconstructed using NUD SPETS software (Nuclear Diagnostics, Stockholm, Sweden) with a modified Shepp–Logan filter and attenuation correction ($\mu = 0.11\ cm^{-1}$) prior to reconstruction (Larsson, 1980). Regions of interest (ROI) were drawn manually on the transversal SPET images using information obtained from the CT scans. An ROI drawn around the tumour area represented tumour tissue and an ROI of similar size on the contralateral side represented normal brain tissue, and an ROI around the superior sagittal sinus represented blood. The background activity was subtracted when calculating the tumour to brain (T:Br), tumor to blood (T:B), and brain to blood (Br:B) ratios from the counts per pixel recorded.

*Blood, urine and tumour samples*

Blood samples were collected at 0, 10, 20 and 40 min and at 1, 2, 3, 4, 7–9, 10–12, 18–21 and 22–25 h after the injection of $^{99m}$Tc-LDL. These data were fitted to the sum of two exponentials as in an earlier study (Vallabhajosula *et al.*, 1988). In one patient the blood time–activity curve was exponential rather than biexponential. Seven patients were subsequently operated on within 22–25 h of administration of $^{99m}$Tc-LDL. Urine samples were collected from two patients between the injection and operation. Radioactivity in blood, urine, and tumour samples (wet weight) was measured with a standard gamma counter (1282 Compugamma, LKB-Wallac, Turku, Finland).

*Statistical analysis*

Group differences were analysed with the chi-square test and correlations were calculated with the least-squares method.

**Results**

In the SPET images $^{99m}$Tc-LDL and $^{99m}$Tc-HSA accumulated in the tumour area as defined by the CT scan (Figures 1–4). The distribution of radioactivity in the tumour areas was not identical for $^{99m}$Tc-LDL and $^{99m}$Tc-HSA, and particularly at 20 h dissimilarities were observed, as can be seen in the three studies presented in Figures 1–3. The tumour to brain (T:Br) ratio increased from 2 h to 20 h (Figure 4) in all patients (Table II). The mean T:Br ratio at 20 h was higher in

**Figure 1** Patient no. 3: previously untreated glioblastoma in right temporal lobe. **a**, CT scan. **b**, $^{99m}$Tc-HSA SPET image at 20 h. **c**, $^{99m}$Tc-LDL SPET image at 20 h.

**Table I** Characteristics of patients with previously untreated and recurrent malignant glioma

| *Patient no.* | *Age (years)* | *Sex* | *Histological diagnosis* | *Location* | *Type of tumour* | *Previous radiotherapy* |
|---|---|---|---|---|---|---|
| 1 | 69 | Male | Glioblastoma | L parieto-occipital | Untreated | No |
| 2 | 29 | Male | Malignant glioma, grade III | L frontal | Untreated | No |
| 3 | 51 | Female | Glioblastoma | R temporal | Untreated | No |
| 4 | 62 | Male | Glioblastoma | R temporoparietal | Untreated | No |
| 5 | 43 | Male | Oligodendroglioma, grade III | R frontotemporoparietal | Recurrent | Yes |
| 6 | 65 | Female | Astrocytoma, grade III[a] | L frontoparietal | Recurrent | Yes |
| 7 | 56 | Female | Oligodendroglioma, grade III | L frontal | Recurrent | Yes |
| 8 | 42 | Male | Astrocytoma, grade III[a] | L frontoparietal | Recurrent | Yes |
| 9 | 43 | Female | Astrocytoma, grade II | R frontal | Recurrent | Yes |

[a]Patient was not considered reoperable; the histological diagnosis was from the initial operation. L, left; R, right.

recurrent tumours than in untreated tumours for both tracers, although the difference was not statistically significant ($P > 0.3$). In recurrent cases the T:Br ratios for radiolabelled LDL and HSA correlated at 20 h, whereas in the untreated cases there was no correlation (Figure 5). The mean tumour to blood (T:B) ratio in untreated tumours was 0.7 for both $^{99m}$Tc-LDL and $^{99m}$Tc-HSA, in recurrent tumours the T:B ratio was 0.9 for $^{99m}$Tc-LDL and 1.2 for $^{99m}$Tc-HSA. The brain to blood (Br:B) ratio remained constant between 2 h and 20 h for both groups.

The mean half-life of the slow component of the $^{99m}$Tc-LDL was 21.5 h (range 14–31); the mean half-life of the fast component was 19 min (range 4–80) (Figure 6). One day after the injection of $^{99m}$Tc-LDL about 35% (range 28–45%) of the injected radioactivity remained in the circulation. Urinary excretion was 7.3% and 10.9% of the injected dose (ID) during the first 24 h in two patients given $^{99m}$Tc-LDL.

The $^{99m}$Tc-LDL activity (per tissue wet weight) in the tumour samples varied from 0.02 to 0.56 mCi g$^{-1}$ (0.09–2.29 $\times$ 10$^{-3}$% ID g$^{-1}$) the mean being 0.27 mCi g$^{-1}$ (1.05 $\times$ 10$^{-3}$% ID g$^{-1}$). There was no correlation between the T:B ratios derived from the SPET images or the T:B ratio determined from the tumour samples.

**Figure 2** Patient no. 4: previously untreated glioblastoma in right temporoparietal area. **a**, CT scan. **b**, $^{99m}$Tc-HSA SPET image at 20 h. **c**, $^{99m}$Tc-LDL SPET image at 20 h.

**Figure 3** Patient no. 5: recurrent anaplastic oligodendroglioma in right frontotemporoparietal area. **a**, CT scan. **b**, $^{99m}$Tc-HSA SPET image at 20 h. **c**, $^{99m}$Tc-LDL SPET image at 20 h.

**Table II** Uptake of radioactivity in gliomas in SPET images after intravenous administration of $^{99m}$Tc-LDL and $^{99m}$Tc-HSA

| | *2 h SPET* | | | | | | *20 h SPET* | | | | | |
|---|---|---|---|---|---|---|---|---|---|---|---|---|
| *Patient* | *T:Br* | | *T:B* | | *Br:B* | | *T:Br* | | *T:B* | | *Br:B* | |
| *no.* | *LDL* | *HSA* | *LDL* | *HSA* | *LDL* | *HSA* | *LDL* | *HSA* | *LDL* | *HSA* | *LDL* | *HSA* |
| 1 | 1.2 | 1.2 | 0.5 | 0.6 | 0.4 | 0.5 | 1.5 | 1.7 | 0.8 | 0.7 | 0.5 | 0.4 |
| 2 | 0.9 | 1.1 | 0.3 | 0.6 | 0.3 | 0.5 | 1.1 | 1.7 | 0.4 | 0.5 | 0.4 | 0.4 |
| 3 | 1.3 | 1.7 | 0.5 | 0.7 | 0.4 | 0.4 | 1.6 | 1.8 | 0.8 | 1.0 | 0.5 | 0.6 |
| 4 | 1.3 | 1.3 | 0.5 | 0.5 | 0.4 | 0.4 | 1.8 | 1.5 | 0.7 | 0.6 | 0.4 | 0.4 |
| Mean | 1.18 | 1.33 | 0.45 | 0.60 | 0.38 | 0.45 | 1.50 | 1.68 | 0.68 | 0.70 | 0.45 | 0.45 |
| 5[a] | 1.5 | 2.3 | 0.6 | 1.2 | 0.4 | 0.5 | 2.7 | 4.8 | 1.0 | 1.8 | 0.4 | 0.4 |
| 6[a] | 0.9 | 1.0 | 0.3 | 0.4 | 0.3 | 0.4 | 1.6 | 1.3 | 0.5 | 0.6 | 0.3 | 0.5 |
| 7[a] | 1.4 | 1.7 | 0.6 | 1.0 | 0.4 | 0.6 | 1.6 | 2.8 | 0.8 | 1.3 | 0.5 | 0.5 |
| 8[a] | 1.4 | ND | 0.6 | ND | 0.4 | ND | 2.6 | ND | 1.2 | ND | 0.5 | ND |
| 9[a] | 1.6 | 1.3 | 0.5 | 0.7 | 0.3 | 0.5 | 2.6 | 4.5 | 1.2 | 1.0 | 0.5 | 0.2 |
| Mean | 1.36 | 1.58 | 0.5 | 0.83 | 0.4 | 0.50 | 2.22 | 3.35 | 0.94 | 1.18 | 0.44 | 0.40 |
| Grand mean ± s.d. | 1.28 ± .24 | 1.45 ± .43 | 0.49 ± .12 | 0.71 ± .26 | 0.37 ± .05 | 0.48 ± .07 | 1.90 ± .58 | 2.51 ± 1.4 | 0.82 ± .28 | 0.94 ± .44 | 0.44 ± .07 | 0.43 ± .12 |

[a]Patients with recurrent, previously operated and radiated tumours. T:Br, tumour to brain ratio; T:B, tumour to blood ratio; Br:B, brain to blood ratio; ND, not determined.

Figure 4 Patient no. 8: recurrent anaplastic astrocytoma in left frontoparietal area. a, CT scan. b, $^{99m}$Tc-LDL SPET image at 2 h. c, $^{99m}$Tc-LDL SPET image at 20 h.

Figure 5 Correlation between tumour to brain radioactivity ratios (T:Br) at 20 h in patients with untreated (●) ($R = 0.202$) and recurrent (▲) ($R = 0.858$) glioma administered LDL and HSA labelled with $^{99m}$Tc.

Figure 6 a, A typical disappearance curve of $^{99m}$Tc-LDL from blood was biexponential in patient no. 1. b, $T_{\frac{1}{2}}$ for the fast component was 8.7 min and for the slow component 22 h.

### Discussion

The metabolism of lipoproteins in gliomas is poorly understood and there is no earlier information on the uptake of LDL in human gliomas *in vivo*, although LDL radiolabelling and metabolism in general has been extensively investigated in humans (Kesäniemi *et al.*, 1983; Lees *et al.*, 1985; Goldstein and Brown, 1989; Lees and Lees, 1991; Virgolini *et al.*, 1991; Leitha *et al.*, 1993). Our observations show that radiolabelled LDL accumulates in gliomas. The uptake of labelled LDL was, however, not higher than that of labelled albumin, a standard blood pool marker, and therefore this study does not provide conclusive evidence of a homogeneous, specific uptake of LDL in gliomas. The observed differences in the distribution of radioactivity in the tumour areas in patients given LDL and HSA indicate, nevertheless, that the mechanism for accumulation of LDL could be different from that of HSA. Albumin, with a molecular weight of 66 kDa, is known to diffuse passively through the disrupted blood–brain barrier (BBB). The molecular weight of LDL is much higher (3 MDa) and therefore the diffusion rate through the disrupted BBB is correspondingly slower, which could explain the somewhat lower T:Br ratio for LDL than for albumin. The rise in the $^{99m}$Tc-LDL T:Br ratio between 2 h and 20 h is probably due primarily to the decrease in blood radioactivity with time, but LDL receptor-mediated uptake may also play a role.

The constant Br:B ratio shows that $^{99m}$Tc-LDL does not cross the intact BBB, and that the radioactivity in the normal brain probably reflects the radioactivity in the circulation. The T:B ratios were quite low in both treated and untreated tumours. However, previous studies conducted with brain phantoms in this laboratory show that a single-head SPET camera underestimates the true target-to-non-target ratio in brain SPET images (Nikkinen *et al.*, 1993). The somewhat higher T:B ratios in recurrent tumours, compared to untreated tumours, are probably due to an additional radiation-induced disruption of the BBB.

Gliomas are known to be very heterogeneous and often contain necrotic and cystic components, explaining why tumour activities determined from SPET scans did not correlate with activities measured from tissue samples. Therefore tissue samples can only be considered representative if taken from a relatively homogeneous tumour, which is not the case with gliomas.

In conclusion, this study shows that the magnitude of $^{99m}$Tc-LDL accumulation in human malignant glioma is similar to that of $^{99m}$Tc-HSA and that the mechanism of LDL uptake may be mostly passive diffusion, in addition to a blood pool effect. However, the different intratumoral distribution of radioactivity in patients given LDL and albumin, along with the different rate of uptake in the tumours, shows that the behaviour of these two substances in gliomas is not identical. Consequently, the uptake of LDL might therefore result from both non-specific and LDL receptor-mediated processes. Further studies on cellular and receptor mechanisms will be needed to elucidate the nature of LDL uptake into human gliomas.

**Acknowledgements**
The authors acknowledge the financial support from the Clinical Research Institute, Helsinki University Central Hospital, Finland.

**References**

BARTH RF AND SOLOWAY AH. (1992). Boron neutron capture therapy for cancer. Realities and prospects. *Cancer*, **70**, 2995-3007.

BROWN MS AND GOLDSTEIN JL (1986). A receptor-mediated pathway for cholesterol homeostasis. *Science*, **232**, 34-47.

CANCER SOCIETY OF FINLAND (1992). Cancer Incidence in Finland 1989 and 1990. *Cancer Statistics of the National Agency for Welfare and Health*. Publication no. 51. Cancer Society of Finland: Helsinki.

DEWIT L, MOSS R AND GABEL D. (1990). New developments in neutron capture therapy. *Eur. J. Cancer*, **26**, 912-914.

DHEW (1974). Lipid and lipoprotein analysis. In *Manual of Laboratory Operations*, Lipid Research Clinics Program, Publication NIH 75-628. DHEW: Washington DC.

FILIPOWSKA D, FILIPOWSKI T, MORELOWSKA B, KAZANOWSKA W, LAUDANSKI T, LAPINJOKI S, ÅKERLUND M AND BREEZE A. (1992). Treatment of cancer patients with a low-density lipoprotein delivery vehicle containing a cytotoxic drug. *Cancer Chemother. Pharmacol.*, **29**, 396-400.

FOGELHOLM R, UUTELA T AND MURROS K. (1984). Epidemiology of central nervous system tumors. A regional survey in Central Finland. *Acta Neurol. Scand.*, **69**, 129-136.

GAL D, OHASHI M, MACDONALD PC, BUCHSBAUM HJ AND SIMPSON ER. (1981). Low-density lipoprotein as a potential vehicle for chemotherapeutic agents and radionucleotides in the management of gyneocologic neoplasms. *Am. J. Obstet. Gynecol.*, **139**, 877-885.

GOLDSTEIN JL AND BROWN MS. (1989). Familial hypercholesterolemia. In *The Metabolic Basis of Inherited Disease*, 6th edn. Scriver CR, Beaudet AL, Sly WS and Valle D. (eds) pp. 1215-1250. McGraw Hill: New York.

HARITZ D, GABEL D AND HUISKAMP R. (1994). Clinical phase-I study of $Na_2B_{12}SH$ (BSH) in patients with malignant glioma as precondition for boron neutron capture therapy (BNCT). *Int. J. Radiat. Oncol. Biol. Phys.*, **28**, 1175-1181.

KAHL SB AND CALLAWAY JC. (1989). New tumor localizers: advances in the use of low density lipoproteins (LDL). *Strahlenther. Onkol.*, **165**, 137-39.

KALLIO M, SANKILA R, JÄÄSKELÄINEN J, KARJALAINEN S AND HAKULINEN T. (1991). A population based study on the incidence and survival rates of 3857 glioma patients diagnosed from 1953 to 1984. *Cancer*, **68**, 1394-1400.

KESÄNIEMI YA, WITZTUM JL AND STEINBECHER UP. (1983). Receptor-mediated catabolism of low density lipoprotein in man. *J. Clin. Invest.*, **71**, 950-959.

LARSSON SA. (1980). Gamma camera emission tomography. *Acta Radiol.* **363** (Suppl.), 1-75.

LASTER BH, KAHL SB, POPENOE EA, PATE DW AND FAIRCHILD RG. (1991). Biological efficacy of boronated low-density lipoprotein for boron neutron capture therapy as measured in cell culture. *Cancer Res.*, **51**, 4588-4593.

LEES AM AND LEES RS. (1991). $^{99m}$Technetium-labeled low density lipoprotein: receptor recognition and intracellular sequestration of radiolabel. *J. Lipid Res.*, **32**, 1-9.

LEES RS, GARABEDIAN HD, LEES AM, SCHUMACHER DJ, MILLER A, ISAACSOHN JL, DERKSEN A AND STRAUSS HW. (1985). Technetium-99m low density lipoproteins: preparation and biodistribution. *J. Nucl. Med.*, **26**, 1056-1062.

LEITHA T, STAUDENHERZ A, GMEINER B, HERMANN M, HÜTTINGER M AND DUDCZAK R. (1993). Technetium-99m labelled LDL as a tracer for quantitative LDL scintigraphy. II. *In vivo* validation, LDL receptor-dependent and unspecific hepatic uptake and scintigraphic results. *Eur. J. Nucl. Med.*, **20**, 674-679.

MCCOMB RD AND BIGNER DD. (1984). The biology of malignant gliomas - a comprehensive survey. *Clin. Neuropathol.*, **3**, 93-106.

MURAKAMI M, USHIO Y, MIHARA Y, KURATSU J-L, HORIUCHI S AND MORINO Y. (1990). Cholesterol uptake by human glioma cells via receptor-mediated endocytosis of low-density lipoprotein. *J. Neurosurg.*, **73**, 760-767.

NIKKINEN P, LIEWENDAHL K, SAVOLAINEN S AND LAUNES J. (1993). Validation of quantitative brain dopamine D2 receptor imaging with a conventional single-head SPET camera. *Eur. J. Nucl. Med.*, **20**, 680-683.

RUDLING MJ, ANGELIN B, PETERSON CO AND COLLINS VP. (1990). Low density lipoprotein receptor activity in human intracranial tumors and its relation to the cholesterol requirement. *Cancer Res.*, **50**, 483-487.

THAKUR ML AND DEFULVIO JD. (1991). Technetium-99m-labeled monoclonal antibodies for immunoscintigraphy. *J. Immunol. Methods*, **137**, 217-224.

THAKUR ML, ESHBACH J, WILDER S, JOHN E AND McDEVITT MR. (1992). Tc-99m labeled sandostatin: preparation and preliminary evaluation (abstract). *IX International Symposium on Radiopharmaceutical Chemistry*, pp. 365-367. Conservatoire National des Arts et Metiers: Paris.

VALLABHAJOSULA S, PAIDI M, BADIMON JJ, LE N-A, GOLDSMITH SJ, FUSTER V AND GINSBERG HN. (1988). Radiotracers for low density lipoprotein biodistribution studies *in vivo*: Technetium-99m low density lipoprotein versus radioiodinated low density lipoprotein preparations. *J. Nucl. Med.*, **29**, 1237-1245.

VIRGOLINI I, RAUSCHA F, LUPATELLI G, ANGELBERGER P, VENTURA A, O'GRADY J AND SINZINGER H. (1991). Autologous low-density lipoprotein labelling allows characterization of human atherosclerotic lesions *in vivo* as to presence of foam cells and endothelial coverage. *Eur. J. Nucl. Med.*, **18**, 948-951.

VITOLS S. (1991). Uptake of low-density lipoprotein by malignant cells: possible therapeutic applications. *Rev. Cancer Cells*, **3**, 488-495.

VITOLS S, ANGELIN B, ERICSSON S, GAHRTON G, JULIUSSON G, MASQUELIER M, PAUL C, PETERSON C, RUDLING M, SÖDERBERG-REID K AND TIDEFELT U. (1990). Uptake of low density lipoproteins by human leukemic cells *in vivo*: relation to plasma lipoprotein levels and possible relevance for selective chemotherapy. *Proc. Natl Acad. Sci. USA*, **87**, 2598-2602.

VITOLS S, PETERSON C, LARSSON O, HOLM P AND ÅBERG B. (1992). Elevated uptake of low density lipoproteins by human lung cancer tissue in vivo. *Cancer Res.*, **52**, 6244-6247.